# Professional Social Media Use Among Orthopedic and Trauma Surgeons in Germany: Cross-Sectional Questionnaire-Based Study

**DOI:** 10.2196/53336

**Published:** 2024-04-19

**Authors:** Yasmin Youssef, Tobias Gehlen, Jörg Ansorg, David Alexander Back, Julian Scherer

**Affiliations:** 1 Department of Orthopaedics, Trauma and Reconstructive Surgery University Hospital Leipzig Leipzig Germany; 2 Center for Musculoskeletal Surgery Charité - Universitätsmedizin Berlin Corporate Member of Freie Universität Berlin and Humboldt-Universität zu Berlin Berlin Germany; 3 Akademie Deutscher Orthopäden Berufsverband für Orthopädie und Unfallchirurgie e.V. Berlin Germany; 4 Dieter Scheffner Center for Medical Education and Educational Research Charité - Universitätsmedizin Berlin Corporate Member of Freie Universität Berlin and Humboldt-Universität zu Berlin Berlin Germany; 5 Department of Traumatology University Hospital of Zurich Zurich Switzerland; 6 Orthopaedic Research Unit University of Cape Town Cape Town South Africa

**Keywords:** social media, digitalization, digital communication, orthopedics, traumatology

## Abstract

**Background:**

Social media (SM) has been recognized as a professional communication tool in the field of orthopedic and trauma surgery that can enhance communication with patients and peers, and increase the visibility of research and offered services. The specific purposes of professional SM use and the benefits and concerns among orthopedic and trauma surgeons, however, remain unexplored.

**Objective:**

This study aims to demonstrate the specific uses of different SM platforms among orthopedic and trauma surgeons in Germany as well as the advantages and concerns.

**Methods:**

A web-based questionnaire was developed on the use of SM in a professional context by considering the current literature and the authors’ topics of interest. The final questionnaire consisted of 33 questions and was distributed among German orthopedic and trauma surgeons via the mail distributor of the Berufsverband für Orthopädie und Unfallchirurgie (Professional Association of Orthopaedic Surgeons in Germany). The study was conducted between June and July 2022. A subgroup analysis was performed for sex (male vs female), age (<60 years vs ≥60 years), and type of workplace (practice vs hospital).

**Results:**

A total of 208 participants answered the questionnaire (male: n=166, 79.8%; younger than 60 years: n=146, 70.2%). In total, all of the participants stated that they use SM for professional purposes. In contrast, the stated specific uses of SM were low. Overall, the most used platforms were employment-oriented SM, messenger apps, and Facebook. Instagram emerged as a popular choice among female participants and participants working in hospital settings. The highest specific use of SM was for professional networking, followed by receiving and sharing health-related information. The lowest specific use was for education and the acquisition of patients. Conventional websites occupied a dominating position, exceeding the use of SM across all specific uses. The key benefit of SM was professional networking. Under 50% of the participants stated that SM could be used to enhance communication with their patients, keep up-to-date, or increase their professional visibility. In total, 65.5% (112/171) of participants stated that SM use was time-consuming, 43.9% (76/173) stated that they lacked application knowledge, and 45.1% (78/173) stated that they did not know what content to post. Additionally, 52.9% (91/172) mentioned medicolegal concerns.

**Conclusions:**

Overall, SM did not seem to be used actively in the professional context among orthopedic and trauma surgeons in Germany. The stated advantages were low, while the stated concerns were high. Adequate education and information material are needed to elucidate the possible professional applications of SM and to address legal concerns.

## Introduction

In the last decades, social media (SM) use has become increasingly popular among all age groups and affects almost all areas of daily life [1-3]. In 2024, the most popular SM platforms worldwide were Facebook, YouTube, WhatsApp, and Instagram [4].

SM has also gained popularity and importance in the health care sector. Regular SM use among health care providers is as high as 88% [5]. In the health care sector, SM can be used by individual physicians as well as health care institutions (hospitals, journals, societies, etc) to facilitate web-based representation as well as communication with patients, potential patients, and the public [6-9]. One advantage of SM is the low barrier for interactions between users and the fast distribution of information and media from almost anywhere at any time. SM can enhance communication with patients and foster professional development, and has the potential to contribute to the spread of (public) health information [10,11]. In the field of surgery, SM has been shown to serve as a tool for research dissemination and surgical education [12,13]. In particular, it could be demonstrated that there is a correlation between the number of SM posts and academic citations of recent research [14,15]. However, while acknowledging those potential advantages of professional SM use, it must be also noted that there are certain dangers, which include violations of patient privacy and medicolegal, confidentiality, and liability issues [7-9,16]. Furthermore, SM incorporates the risk of spreading health-related misinformation [17,18].

Recent studies have shown that the rate of SM use among orthopedic and trauma surgeons lies between 37% to 100% [19-25]. However, the specific uses of SM in a professional context and the barriers toward comprehensive use within the field of orthopedics and trauma surgery remain unknown. In addition to that, data on the appreciated advantages and concerns of professional SM use in the field of orthopedic and trauma surgery is lacking.

Thus, this study aimed to assess the appreciated advantages and concerns of professional SM use among orthopedic and trauma surgeons in Germany. Further, we aimed to present the current professional use of different SM platforms used by orthopedic and trauma surgeons in Germany.

## Methods

### Study Design

A web-based questionnaire was created to assess the current use of SM among orthopedic and trauma surgeons in Germany. We used SurveyMonkey (SurveyMonkey Inc), web-based software for creating questionnaires. The link to the questionnaire was shared via the mail distributor of the Berufsverband für Orthopädie und Unfallchirurgie (Professional Association of Orthopaedic Surgeons in Germany). The study was conducted between June and July 2022.

### Ethical Considerations

Participation in the web-based questionnaire was voluntary and anonymous, with no identifying data collected, except for age, gender, and occupation. Anonymous questionnaires, by definition, do not collect personal data that can directly or indirectly identify an individual. This means that such surveys do not fall under the scope of the European General Data Protection Regulation (GDPR), as the GDPR only applies to the processing of personal data (Article 5) [26]. Additionally, the presented survey does not require a Data Protection Impact Assessment according to Article 35 of the GDPR [26]. No formal ethical approval by an ethics committee was needed for the study conduction as general waivers apply for surveys with anonymous data in Germany. All participants received written information about the aim and scope of the study as well as how data is collected, processed, and analyzed in the form of a disclaimer before starting the questionnaire (Multimedia Appendix 1). Patients were informed that by answering the questionnaire, they consented to data collection, processing, and use for publication. No incentives were offered for the completion of the questionnaire.

### Questionnaire

The development of the questionnaire was described in a preceding publication [25]. The questionnaire was developed by the study team based on a review of the current literature [19,20,22,24,27-29] and was complemented with further areas of interest. The preliminary and digitalized questionnaire was pretested among 5 orthopedic and trauma surgeons. The questionnaire was finalized considering the feedback from the pilot group. The questionnaire (Multimedia Appendix 1) consisted of 33 variables and included two separate sections. A 5-point Likert scale (where 1 was “I strongly disagree” and 5 was “I strongly agree”) was used to assess the advantages and concerns stated regarding professional SM use.

The first publication highlighted the types of SM platforms used for private and professional purposes, the use behavior, and content management of orthopedic and trauma surgeons in Germany [25]. The second section of this study, which includes questions on the specific uses of different SM platforms as well as the appreciated benefits and concerns, will be analyzed.

### Data Processing and Statistical Analysis

Statistical analysis was performed using SPSS for Mac (version 26.0; IBM Corp). Both complete and incomplete questionnaires were considered in the final data analysis. Categorical data were presented in frequencies and percentages. Subgroup analysis was performed for sex (male vs female), age (<60 years vs ≥60 years), and type of workplace (practice vs hospital). To assess differences between groups, the chi-square test was used for categorical data. Likert scales were compared using a nonparametrical median test. The level of statistical significance was set at a 2-sided *P* value <.05.

## Results

### Demographics

The sample is identical to that previously published [25]. In total, 208 participants took part in this survey (male: n=166, 79.8%), of which 70.2% (n=146) were aged <60 years. Most of the participants (n=161, 77.4%) worked in a practice. More female participants were younger than 60 years (<60 years: 39/42, 92.9% vs ≥60 years: 3/42 4.8%; *P*<.001), and younger participants were more likely to be working in a hospital rather than in a practice (<60 years: 39/47, 83% vs ≥60 years: 8/47, 17%; *P*=.03). Significantly more male participants were in a practice (male: 138/161, 85.7% vs female: 23/161, 14.3%; *P*<.001). As shown in the preceding study, all participants (200/200, 100%) stated that they used SM for professional purposes [25].

### SM for Professional Networking

For professional networking (communication with colleagues), the most used platforms were messenger apps (58/173, 33.5%), employment-oriented SM (47/173, 27.2%), and Facebook (12/173, 6.9%). In addition to that, 39 (22.5%) participants stated that they used conventional websites for communication with colleagues. Female participants were more likely to use Facebook (male: 7/142, 4.9% vs female: 5/31, 16%; *P*=.03) and Instagram (male: 2/142, 2.8% vs female: 4/31, 13%; *P*=.02). Participants working in hospital were more likely to use Facebook (hospital: 6/34, 18% vs practice: 6/139, 4.3%; *P*=.006), Instagram (hospital: 4/34, 12% vs practice: 4/139, 2.9%; *P*=.03), and employment-oriented SM (hospital: 16/34, 47% vs practice: 31/139, 22.3%; *P*=.004).

### SM for Receiving and Sharing Health-Related Information

The platforms used most frequently for receiving health-related information were YouTube (46/174, 26.4%), employment-oriented SM (28/174, 16.1%), and messenger apps (23/174, 13.2%). More than half of the participants (113/174, 64.9%) used conventional websites. Instagram was used more by female participants (male: 4/143, 2.8% vs female: 5/31, 16%; *P*=.002). Participants working in a hospital were more likely to use TikTok (hospital: 1/34, 3% vs practice: 0/0, 0%; *P*=.04). The most often used platforms for sharing health-related information were messenger apps (45/172, 26.2%), employment-oriented SM (23/172, 13.4%), Facebook (10/172, 5.8%), and Instagram (10/172, 5.8%). Conventional websites were only used by a minority of participants (39/172, 22.7%). More female participants used Instagram (male: 5/141, 3.5% vs female: 5/31, 16%; *P*=.007) and TikTok *(*male: 0/142, 0% vs female: 1/31, 3%; *P*=.03). Participants working in the hospital were more likely to use Facebook (hospital: 5/34, 15% vs practice: 5/138, 3.6%; *P*=.01) and TikTok (hospital: 1/34, 3% vs practice: 0/138, 0%; *P*=.04).

### SM for Sharing Clinical Expertise

Messenger apps were used by 21.8% (37/170) of the participants for sharing clinical expertise and professional skills. Employment-oriented SM was used by 12% (22/170) and Instagram by 4.7% (8/170) of participants. In total, 24.7% (42/170) used conventional websites. Female participants were more likely to use Facebook (male: 3/139, 2.2% vs female: 3/31, 10%; *P*=.04) and Instagram (male: 4/139, 2.9% vs female: 4/31, 13%; *P*=.02). Instagram was also used more by younger participants (<60 years: 8/116, 6.9% vs ≥60 years: 0/54, 0%; *P*=.048). In total, 5.3% (9/169) of participants used Facebook, 4.1% (7/169) used employment-oriented SM, and 4.1% (7/169) used messenger apps for producing content on diseases and treatment methods. Overall, 26.6% (45/169) used conventional websites for sharing content on diseases and treatment methods.

### SM for Educational Purposes

The SM platforms used mostly for educational purposes were employment-oriented platforms (eg, LinkedIn; 26/171, 15.2%), YouTube (20/171, 11.7%), and messenger apps (19/171, 11.1%). Conventional websites were used by 60.8% (104/171) of participants. Older participants were significantly more likely to use messenger apps (<60 years: 8/117, 6.8% vs ≥60 years: 11/54, 20%; *P*=.009).

### SM for the Acquisition of and Communication With (Potential) Patients

Only a minority of the participants stated that they use SM to acquire new patients. In total, 8.3% (14/168) of participants used Facebook, 5.4% (9/168) used messenger apps, and 4.2% (7/168) used Instagram. In contrast, 42.9% (72/168) used conventional websites. Overall, messenger apps were significantly used more often by older participants (aged <60 years: 13/118, 11% vs aged ≥60 years: 16/53, 30%; *P*=.002). A further 5.3% (9/171) of participants used Facebook and 4.1% (7/171) used Instagram. Websites were used by 34.5% (59/171) of participants.

### Stated Advantages

In total, 26.6% (47/177) of participants agreed or strongly agreed that professional SM use could help them in the acquisition of new patients. In contrast, 28.8% (51/177) of participants adopted a neutral position, and 44.6% (79/177) disagreed or disagreed strongly. Similarly, only 24.3% (43/177) of participants agreed or strongly agreed that professional SM use could improve their communication with patients, while 53.1% (94/177) disagreed or strongly disagreed with this statement. Additionally, 42.4% (75/177) of participants agreed or strongly agreed that SM could help them stay up-to-date professionally, and 40.1% (71/177) agreed that SM could help them to increase their visibility. There were no significant differences found for age, gender, and type of workplace. The median values are presented in Table 1.

**Table 1:** Stated advantages of professional social media use by German orthopedic and trauma surgeons (n=177).

	Score^a^, median (IQR)
Social media helps me acquire new patients	3.00 (1.63-4.38)
Social media helps me communicate with my patients	4.00 (3.00-5.00)
Social media help me stay up-to-date professionally	3.00 (2.00-4.00)
Social media helps me represent my medical offers	3.00 (1.50-4.50)

^a^Likert scale where 1 is “I fully disagree” and 5 is “I fully agree.”

### Stated Concerns

In total, 65.5% (112/171) of participants agreed or strongly agreed that the professional use of SM was time-consuming. Only 8.8% (15/171) of participants disagreed or strongly disagreed with this statement. Overall, 45.1% (78/173) of participants agreed or strongly agreed that they have difficulties assessing what content would interest patients on SM. Furthermore, 52.9% (91/172) of participants agreed or strongly agreed that they were insecure with the medicolegal and data protection regulations concerning SM, while only 25.6% (44/172) disagreed or strongly disagreed.

In addition, 43.9% (76/173) of participants agreed or strongly agreed that they lacked knowledge on the use of SM. In total, 31.2% (54/173) of participants stated that they disagreed or strongly disagreed with this statement. Older participants stated more often that they lack knowledge of SM implementation and use *(P*=.006). The median values are presented in Table 2.

**Table 2:** Stated concerns about professional social media use by German orthopedic and trauma surgeons.

	Score^a^, median (IQR)
The use of social media is too time-consuming (n=171)	2.00 (1.00-3.00)
I don’t have sufficient knowledge on the efficient use of social media in the workplace (n=173)	3.00 (2.00-4.00)
I am insecure with legal and data protection regulation (n=172)	2.00 (1.00-3.00)
I find it hard to estimate which content interests patients (n=173)	3.00 (2.00-4.00)

^a^Likert scale where 1 is “I fully disagree” and 5 is “I fully agree.”

## Discussion

### Principal Findings

In the health care system, SM is shaping the ways and dimensions of communication and the dissemination and consumption of (health) information [7,30]. The active use and popularity of SM in the professional context was particularly accelerated by the COVID-19 pandemic [31,32]. Several benefits of professional SM use have been previously identified. SM can enable fast and location-independent sharing of health-related information, which makes it more accessible to health care professionals, patients, and the public [6-8,30,33-35]. Furthermore, SM can improve communication within the health care system and increase the visibility of individual physicians, institutions, or publishing companies [14,15].

SM is also shaping the field of orthopedic and trauma surgery [29,36]. Several studies have investigated the prevalence of professional SM use among orthopedic and trauma surgeons and have reported rates of professional SM use between 37% to 65.7% among the assessed populations [19-24]. In Germany, a recent study showed that 100% of participants used SM for professional purposes. A structured implementation into daily professional work routines, however, seemed to be lacking [25]. Overall, employment-oriented SM like LinkedIn, Facebook, and YouTube are among the most used platforms [19,20,22,24,27,36]. There is, however, limited data concerning the specific use and application of SM and the appreciated benefits and concerns of SM among orthopedic and trauma surgeons.

Overall, the most prevalent use of SM in the professional context of the presented cohort was for professional networking. Almost one-third of the participants used messenger apps and employment-oriented SM for professional networking. Other SM like Facebook, Instagram, and Twitter on the other hand seemed to play a minor role. This low use of SM for professional networking was not expected, as a previous study by Grossman et al [12] highlighted that SM use by surgeons plays an increasingly important role in the dissemination and communication of research results and professional information with colleagues and the public. The sharing of information can, in turn, increase the visibility of a particular surgeon or their research field [14,15]. Furthermore, a study by Justiana et al [37] among 165 Saudi Arabian orthopedic surgeons showed that most of the assessed surgeons used SM for sharing medical knowledge (79.03%), discussing cases with colleagues (72.4%), and sharing work experiences (66.7%). In contrast to this study, Justiana et al [37] did not differentiate between different SM platforms. Analyzing different aspects of professional networking, we found that the most used platforms for receiving health-related information were YouTube, employment-oriented SM, and messenger apps. On the other hand, the most frequently used platforms for sharing health-related information were messenger apps, employment-oriented SM, Facebook, and Instagram. Similarly, messenger apps, employment-oriented SM, and Instagram were the platforms used the most for sharing clinical expertise and professional skills. For the above specific uses, Instagram seemed to be used more by female participants, and TikTok was used more often by participants working in a hospital.

The use of SM for both receiving and sharing health-related information and clinical expertise as well as professional skills was low within the presented study population. In contrast, conventional websites occupied a predominant role for those uses.

The presented results, therefore, suggest that the maximum potential of SM in the dissemination of health-related and clinical information and expertise is currently not being reached.

The use of SM for educational purposes also seemed subsidiary in the presented cohort, while conventional websites were used by more than 60% of the participants for this purpose. In this study, employment-oriented SM, YouTube, and messenger apps, which were the SM platforms used the most for educational purposes, were each used by less than 25% of the participants. In contrast, a study by Schneider et al [31] found among a group of 312 orthopedic residents and medical students that the majority showed a high interest in accessing educational information via SM [31]. This difference may be explained by the fact that the presented study included participants from all age groups and experience levels, and not only residents or medical students who might show a higher affinity to SM due to their younger age. No significant difference could be found for the type and prevalence of SM platforms used for educational purposes. The results suggest that there might still be potential for both providers and consumers of educational offers to actively use and develop educational offers via SM. Previous studies have highlighted that the benefits include the connection of learners independently of time and location, and the opportunity to share and actively react to medical information [38-40].

In this study, SM also seemed to play a subordinate role in the communication and acquisition of patients. The most used platforms for these purposes were messenger apps, Facebook, and Instagram. Overall, 17% stated that they use messenger apps for communication with their patients. All other platforms were used by less than 9% of the participants for acquisition of and communication with patients. On the other hand, conventional websites were used by 34.5% and 42.9%, respectively, for those purposes. While comparable data from the field of orthopedic and trauma surgery is missing, a study among 500 US plastic surgeons showed that almost 50% used SM for marketing purposes and 28% acquired patients via SM [41]. However, it must be noted that the study was published in 2013, and plastic surgeons might rely more on SM as they provide private services in most cases. Similarly, in a survey by Brown et al [42] among Australian doctors, only 1 of 187 respondents used SM to communicate with patients and only 21.2% believed it would be appropriate to do so. Of note, the study was published in 2014 and did not clearly distinguish between private and professional SM profiles, so the comparability is limited. A more recent study by Justiana et al [37] showed a higher use of SM for interacting with patients; it was not differentiated between different SM platforms. Duymus et al [43] reported that 61.6% had concerns about communication with patients through SM. Nonetheless, these results suggest that the use of SM for acquisition of and communication with patients is still not yet implemented among orthopedic surgeons. This could be due to the continuing uncertainty concerning medicolegal issues. Further studies are needed to quantify the impact of SM use on patient acquisition.

Furthermore, these results showed that the stated advantages of professional SM use were low. Only the minority agreed that SM could help them in the acquisition of and communication with patients. No clear trend was seen for the stated benefit regarding increased visibility. However, most participants believed that SM could help them stay up-to-date professionally. To the best of our knowledge, this is the first study that evaluated the attitudes of German orthopedic surgeons toward professional SM use. Comparative data in the field of orthopedics and trauma surgery is missing.

Lastly, most participants had concerns about the professional use of SM. This included expenditure of time and an unawareness of what content is of interest to patients. In addition to that, more than half of the respondents had concerns about medicolegal and data protection issues. Similarly, in a study by Brown et al [42], more than 65% of the participants stated that they had concerns about engaging more on SM due to public access and legal concerns. This might be explained by the fact that there is only limited informative and educational material and tools for the professional use of SM. Additionally, there are no clear regulations for the professional use of SM in Germany. For example, whether a patient’s fully anonymized x-ray can be posted online without the patient’s written approval is still a gray area. In the study by Justiana et al [37], over 50% of the respondents had concerns about legal issues related to interaction with patients, and only 42.5% agreed that it was ethically acceptable to anonymously discuss a patient’s case on SM [37]. The appreciated concerns of the presented cohort correspond to the risks and dangers described in the literature—high time requirement, violation of patients’ anonymity, decrease of professionalism, and legal issues [8,44]. Supporting information material and courses provided by political or health institutions or orthopedic and trauma societies could help orthopedic and trauma surgeons understand how they can safely exploit the full advantages of SM in a professional and legal context.

Overall, these results suggest that SM use among German orthopedic and trauma surgeons is predominantly passive. While all participants stated that they use SM for professional purposes, only a minority indicated concrete uses. In a professional context, websites still play an important role. Professional networking was identified as the key benefit of SM. Overall, the most used platforms were employment-oriented SM, messenger apps, and Facebook. This corresponds to previously presented literature that has shown that employment-oriented SM, Facebook, and YouTube are the most used platforms among orthopedic surgeons [19,20,22,24,27,36].

### Limitations

This study has certain limitations. First, surveys have minor levels of evidence in general, and their outcome can be affected by the participants’ understanding of the questions. Further, the number of participants cannot be taken as representative of all orthopedic and trauma surgeons in Germany. Hence, these results must be treated with caution. Additionally, there were considerable differences in the subgroup sizes that could have impaired the statistical analysis. Furthermore, due to the voluntary nature of participation, orthopedic and trauma surgeons with a more critical attitude toward SM use for professional purposes might be underrepresented, posing a potential bias. Further, the questionnaire itself did not undergo probabilistic theory testing, which might represent further bias.

### Conclusions

The professional use of SM among the assessed participants of German orthopedic and trauma surgeons is predominantly passive. Only a minority produce their own content. Additionally, the stated advantages of professional SM use were low, while the stated concerns were high. The use of SM for professional purposes seems to play a minor role among orthopedic and trauma surgeons. Adequate education and information material on the professional use of SM is needed to potentially elucidate its applications and benefits, and to address legal concerns. Further studies are needed to validate the described trends.

## Appendix

Multimedia Appendix 1 Questionnaire (English translation).

## Abbreviations

GDPR: General Data Protection Regulation
SM: social media

## Data Availability

The data sets generated or analyzed during this study are available from the corresponding author upon reasonable request.
