# Supplementary material for: Professional Social Media Use Among Orthopedic and Trauma Surgeons in Germany: Cross-Sectional Questionnaire-Based Study
Source: JMIR Form Res. 2024 Apr 19;8:e53336. doi: 10.2196/53336 (PMC11069096; doi:10.2196/53336)
Supplement: Multimedia Appendix 1 [file formative_v8i1e53336_app1.docx]

Questionnaire on the use of Social Media among orthopaedic and trauma surgeons in Germany

# Disclaimer

Dear colleagues,

Social media has become an integral part of people's private and professional lives, especially since the introduction of Web 2.0. Physicians, as well as patients, use social media as a means of communication and information.

With this brief survey, we aim to capture your professional use and attitude towards social media in your daily work: Do you use social media as an orthopedist or trauma surgeon, and if so, to what extent and in what way?

Participation in this survey is voluntary and anonymous. By participating, you simultaneously give us your consent for a scientific evaluation and publication of the anonymized data.

We thank you in advance for your commitment and participation!

Sincerely,

PD Dr. med. David Back

Dr. med. Jörg Ansorg

Dr. med. Tobias Gehlen

cand. med. Yasmin Youssef

# Sociodemographic Data

1. Gender:
   - Male
   - Female
   - Diverse
2. Age:
   - < 30
   - 31-40
   - 41-50
   - 51-60
   - > 60
3. Localization of your workplace:
   - Large city (> 100,000 inhabitants)
   - Middle sized town (population between 20,000 and 100,000 inhabitants)
   - Small town (Population < 20,000 inhabitants)
   - Rural community
4. Level of professional training:
   - Resident
   - junior consultant
   - consultant
   - senior consultant
   - consultant in practice
5. In what type of your workplace do you work?
   - medical center
   - Practice
   - hospital
6. How many employees work in your workplace/department?
   - 1-4
   - 5-10
   - >10
   - >20
   - >30
   - >40

# Type of Media used

1. Which social media do you use for private purposes? (multiple answers possible)
   - Twitter
   - Facebook
   - Instagram
   - TikTok
   - employment oriented social networks such as LinkedIn or Xing
   - Messenger Apps (WhatsApp, Telegram, Signal etc.)
   - YouTube
   - Other (please specify)
   - None of the above
2. Which social media do you personally use in your professional environment? (multiple answers possible)
   - Twitter
   - Facebook
   - Instagram
   - TikTok
   - employment oriented social networks such as LinkedIn or Xing
   - Messenger Apps (WhatsApp, Telegram, Signal etc.)
   - YouTube
   - Other (please specify)
   - None of the above
3. What other digital media do you use professionally? (multiple answers possible)
   - podcast
   - blog
   - website
   - Waiting room TV
   - Other (please specify)
   - None of the above

# Management of professional social media accounts and usage behavior

1. Who is in your practice/department is responsible for updating content on your professional social media accounts (e.g. new posts or replying to patient inquiries) (multiple answers possible)
   - Myself
   - Other internal medical staff
   - Other internal non-medical personnel
   - External Vendors
   - Other (please specify)
   - None of the above
2. Do you have separate social media accounts for professional and private purposes?
   - Yes
   - No
3. Have you ever attended a course on the topic of social media for Professionals
   purposes?
   - Yes
   - No
4. How frequently do you use social media for professional purposes?
   - Daily
   - Several times a week
   - Once a week
   - Weekends only
   - At least once a month
   - Infrequently
   - Never
5. I produce own content on diseases/treatment methods.
   - Yes
   - No
6. Do you use your professional social media accounts during work hours?
   - Yes
   - No
7. Do you check how many followers you have on your professional social media account?
   - Yes
   - No
8. If so, how do you react to changing follower numbers?
   - When the number of followers is low, I increase my post frequency
   - If I have a high number of followers, I will keep my post frequency
   - I do not match my post frequency with my follower count
   - Other (please specify)
   - None of the above

# Professional uses of social media

1. For what professional purposes do you use the following social media channels?

|  | Facebook | Twitter | Instagram | TikTok | Employment oriented social media | Messenger Apps | YouTube | Website |
| --- | --- | --- | --- | --- | --- | --- | --- | --- |
| For conducting further training (also CME...). |  |  |  |  |  |  |  |  |
| For receiving health-related information. |  |  |  |  |  |  |  |  |
| For professional networking (networking with colleagues). |  |  |  |  |  |  |  |  |
| For sharing health-related information. |  |  |  |  |  |  |  |  |
| For sharing own clinical experience and expertise. |  |  |  |  |  |  |  |  |
| To produce and post your own content about diseases and treatment methods. |  |  |  |  |  |  |  |  |
| To communicate with patients. |  |  |  |  |  |  |  |  |
| To acquire new patients or to keep existing ones. |  |  |  |  |  |  |  |  |

# Perceived advantages of social media uses

1. What advantages do you see in the use of social media in a professional context?

|  | Strongly agree | agree | Neutral | Disagree | Strongly disagree |
| --- | --- | --- | --- | --- | --- |
| Social media help me acquire patients for my practice/department. |  |  |  |  |  |
| Social media help me communicate and interact with my patients (e.g. for receiving and responding to patient inquiries, criticism and opinions). |  |  |  |  |  |
| Social media help me to keep up to date with the latest developments in my field. |  |  |  |  |  |
| Social media help me to present the offers of my practice/department. |  |  |  |  |  |

# Perceived difficulties of social media use

1. What difficulties do you see in the use of social media in a professional context?

|  | Strongly agree | agree | Neutral | Disagree | Strongly disagree |
| --- | --- | --- | --- | --- | --- |
| Using social media for professional purposes is time-consuming. |  |  |  |  |  |
| I have insufficient knowledge on how to use social media efficiently for professional purposes. |  |  |  |  |  |
| I feel unsure about legal regulations regarding data protection when using social media for professional purposes. |  |  |  |  |  |
| I find it difficult to assess which content on social media appeals to patients. |  |  |  |  |  |
